# Moderating effect of social support in the relationship between perceived work overload and patient safety behaviours among nursing interns in Nigeria

**DOI:** 10.3389/frhs.2025.1703926

**Published:** 2026-01-09

**Authors:** Anthony Gbenro Balogun, Victor Chidi Onyencho, Choja Akpovire Oduaran

**Affiliations:** 1 Department of Pure and Applied Psychology, Adekunle Ajasin University, Ondo, Nigeria; 2 Community Psychosocial Research, North-West University, Potchefstroom, South Africa

**Keywords:** coworker support, JD-R model, nursing interns, patient safety behaviours, supervisor support, work overload

## Abstract

**Background:**

Patient safety-related adverse events continue to pose a serious threat in healthcare, frequently arising from excessive job demands on frontline staff. It is particularly critical to understand how work overload affects nursing interns, a group vulnerable due to limited clinical experience.

**Purpose:**

This study examines the relationship between work overload and patient safety behaviours among nursing interns in Nigerian public hospitals. It also investigates whether perceived supervisor and coworker support moderate that relationship, guided by the Job Demands–Resources (JD-R) model.

**Methods:**

A cross-sectional survey was administered to nursing interns during clinical placements in government teaching hospitals located in the Southwest region of Nigeria, measuring self-reported work overload, perceived supervisor and coworker support, and medical error incidence.

**Results:**

Higher levels of reported work overload were found to significantly predict a higher incidence of self-reported patient safety errors. However, both perceived supervisor support and coworker support significantly moderated this association, helping to buffer the negative impact of work overload on the occurrence of patient safety errors.

**Conclusions:**

Social support from supervisors and colleagues serves as a protective resource in high-stress clinical environments. Health institutions should therefore promote supportive supervisory practices and team cohesion to mitigate patient safety-related adverse events and enhance the well-being and performance of early-career nurses.

## Introduction

Patient safety adverse events remain a critical public health concern globally, with far- reaching consequences for patient safety, healthcare costs, and professional well-being (1, 2). They are commonly defined as preventable adverse events or failures in planned healthcare actions that may result in harm or the potential for harm to patients (3). Such errors encompass medication mistakes, procedural lapses, documentation failures, and communication breakdowns, issues that collectively undermine the quality and safety of care (4, 5).

In this study, patient safety behaviours are conceptualized and measured following previous research that operationalized errors as self-reported lapses in patient safety practices, near misses, or deviations from safe care protocols [e.g., (6–8)]. This approach aligns with widely used self-report scales designed to capture healthcare providers' engagement in or avoidance of error-prone behaviors and adherence to patient safety standards (56). Although self-reported measures may reflect perceptions of safety rather than objectively verified incidents, prior work shows they are reliable proxies for understanding underlying behavioral and organizational factors that lead to patient safety behaviours (3, 9).

Empirical evidence consistently links excessive workload to increased risk of patient safety behaviours (10). For instance, Dolev et al. (11) found that high job demands and long working hours significantly predict error occurrence and compromised patient safety. Similarly, a recent large-scale analysis (12) demonstrated that higher workloads were associated with more frequent incident of medication errors among nurses. Furthermore, Im et al. (57) found that higher levels of psychological burnout, shorter meal breaks during duty hours, and longer weekly overtime were significantly associated with an increased likelihood of medication errors among nurses working in tertiary university hospitals. These findings underscore the central role of workload as a system-level determinant of safety outcomes. However, while such relationships are well-documented among experienced nurses and physicians, the extent to which workload contributes to patient safety-related errors among nursing interns, a group characterized by limited clinical experience and transitional stress, remains poorly understood (8, 9, 13, 14).

This issue is particularly salient in low-resource settings like Nigeria, where chronic workforce shortages, inadequate supervision, and heavy patient loads create conditions conducive to work overload (15, 16). Nursing interns, who are still developing their clinical judgment and procedural confidence, often face substantial workload pressures that may increase the likelihood of patient safety incidents (17). Yet, empirical investigations addressing this issue within the Nigerian context remain limited.

Moreover, emerging evidence indicates that social support from supervisors and coworkers can mitigate the adverse effects of workload on performance and safety (18). In high-pressure clinical settings, adequate social support can reduce stress, strengthen coping strategies, and foster resilience, thereby lowering the likelihood of patient safety incidents among nursing interns. Conversely, insufficient support may intensify the negative effects of work overload, making interns more susceptible to mistakes. Although prior studies have examined related constructs (13, 14), no empirical study has specifically investigated the moderating role of social support in the relationship between work overload and patient safety behaviours among nursing interns.

Given the significant implications of patient safety-related errors and the limited empirical focus on nursing interns in Nigeria, it is essential to examine how work overload influence patient safety behaviours and whether social support moderates this relationship. Understanding these relationships will provide valuable insights for healthcare administrators, policymakers, and educators to design targeted interventions that reduce workload pressures, strengthen social support systems, and ultimately enhance both patient safety and the professional development of nursing interns.

Therefore, using the job demands–resources (JD-R) model (19) as a theoretical framework, the present study addresses this gap by examining the moderating role of social support (supervisor and coworker support) in the relationship between work overload and patient safety behaviours among a sample of nursing interns in Nigeria. This study contributes to the literature in three key ways. First, it advances understanding of how work overload influences patient safety behaviours in a developing-country context, focusing on a population that is underrepresented in empirical research yet plays a crucial role in healthcare delivery. Second, it extends the JD-R model by testing the moderating effects of supervisor and coworker support in a Sub-Saharan African healthcare environment. Third, it provides context- specific insights with practical implications for healthcare policy, internship program design, and workplace interventions aimed at improving patient safety. By highlighting the protective roles of supervisor and coworker support, this study underscores the importance of fostering supportive clinical environments as a strategic approach to reducing patient safety-related errors and safeguarding the well-being of early-career nurses.

## Nursing internship context in Nigeria

In Nigeria, the nursing internship is a structured, 12-month clinical program that serves as a transition from nursing education to professional practice (20). During this period, interns are expected to rotate through multiple hospital units, gaining exposure to diverse clinical settings while providing direct patient care under supervision (21). Typical workloads for nursing interns are often high, with extended shifts, frequent night duties, and simultaneous responsibilities across units. Compounding these demands, staff shortages are prevalent in many Nigerian healthcare facilities, particularly in public and low-resource hospitals, resulting in increased patient-to-nurse ratios and additional clinical responsibilities for interns (22). These structural and systemic factors create a challenging work environment that may influence both perceived stress and the likelihood of errors in patient care (17, 23), underscoring the relevance of examining work overload and patient safety behaviours within this population.

## Literature review and hypotheses development theoretical background

As mentioned earlier, the present study draws upon the JD-R model (19) as its primary theoretical framework. The JD-R model posits that every occupation has specific risk factors associated with job stress, categorized broadly into job demands and job resources. Job demands refer to those physical, psychological, social, or organizational aspects of the job that require sustained effort and are associated with certain physiological and psychological costs (e.g., work overload). In contrast, job resources are those physical, psychological, social, or organizational aspects that help achieve work goals, reduce job demands, and stimulate personal growth (e.g., supervisor and coworker support) (24).

Within this framework, work overload is conceptualized as a key job demand that places excessive pressure on nursing interns, leading to fatigue, reduced cognitive functioning, and heightened susceptibility to patient safety-related adverse events. Patient safety adverse events, in turn, can have serious consequences, not only for patients but also for the psychological well- being and professional development of nursing interns.

Crucially, the JD-R model introduces the concept of buffering, wherein job resources mitigate the adverse effects of job demands on work-related outcomes (19). In this study, perceived supervisor support and coworker support are positioned as vital job resources capable of buffering the positive relationship between work overload and patient safety errors. According to JD-R model, supervisor and coworker support can enhance coping mechanisms, foster resilience, and provide practical and emotional resources that enable interns to manage overwhelming workloads more effectively (19, 25), thus reducing the likelihood of errors.

## Perceived work overload and patient safety behaviours

Work overload arises when job demands exceed an individual's available resources and capabilities (26). Employees experiencing work overload frequently report feeling overwhelmed by excessive responsibilities and perceive a lack of sufficient time or resources to manage their tasks effectively (27). In healthcare settings, work overload is widely recognized as a significant occupational stressor, often linked to adverse outcomes such as burnout, emotional exhaustion, and impaired job performance (28). According to Zheltoukhova (29), workload refers to work demands that surpass personal capacities, eliciting psychological responses such as nervousness, anxiety, frustration, and irritation.

Within the nursing profession, work overload has been associated with reduced cognitive functioning, diminished attentiveness, and an elevated risk of patient safety behaviours (11). Nursing interns, in particular, face substantial work demands without the benefit of extensive experience or developed coping mechanisms, which may heighten their susceptibility to clinical errors and compromise patient safety (30).

Evidence from various healthcare systems, including those in high-income countries, has demonstrated a strong link between excessive workloads and increased patient safety incidents (31). For instance, Abdul Salam et al. (32) reported that work-related stress, particularly excessive workload, was significantly associated with medication error. Similarly, Carrez et al. (33) found a positive relationship between work overload and the likelihood of errors during chemotherapy preparation. In a related study, Ratanto, Hariyati, Mediawati, and Eryando (12) concluded that work overload was the most influential factor contributing to medication errors, surpassing the effects of work experience, motivation, managerial support, and environmental conditions. Furthermore, Kasalak et al. (34) observed that diagnostic errors among radiologists were often preceded by relative work overload, particularly during the interpretation of CT scans.

Despite growing international evidence, research on the relationship between work overload and patient safety behaviours remains scarce in the Nigerian context, where systemic issues such as understaffing and limited resources may intensify the impact of excessive work demands, especially on nursing interns. In light of these challenges and drawing on the JD-R model, the following hypothesis is proposed:

*H1*: Work overload is positively associated with patient safety behaviours among nursing interns.

## Perceived supervisor support as a buffer

Grounded in the JD-R model (35), the present study posits that perceived supervisor support can mitigate the detrimental effects of work overload on patient safety behaviours. Within the JD-R framework, perceived supervisor support is considered a key job resource, a social mechanism that can buffer the negative impact of high job demands (19). Supervisor support encompasses emotional encouragement, instrumental assistance, constructive feedback, and recognition, all of which contribute to a psychologically safe and resourceful work environment (58).

According to the JD-R model, excessive job demands, such as work overload, can deplete an employee's physical and mental resources, leading to fatigue, reduced cognitive capacity, and stress, which in turn elevate the risk of performance failures, including patient safety-related adverse events (19). However, the availability of supervisor support can act as a protective buffer, moderating the adverse effects of overload by enhancing coping capacity, psychological safety, and task prioritization (28).

Empirical studies have lent support to this buffering effect. For example, Linde et al. (36) found that medical residents who perceived high supervisor support reported lower incidences of patient safety behaviours. Similarly, Gorgich et al. (37) identified adequate supervision and support as key factors in reducing medication errors among nurses. Seo and Lee (38) further demonstrated that when hospital management and supervisors foster a strong safety culture and provide necessary resources, nurses are more inclined to engage in speaking- up behaviors, which reduces the likelihood of clinical mistakes. Additionally, Liu et al. (39) showed that daily fluctuations in supervisor support were directly associated with corresponding changes in service performance, mediated through daily positive affect.

Several other studies reinforce the moderating role of supervisor support in high-demand settings. For example, Weigl et al. (40) found that supervisor support buffered the relationship between emotional exhaustion and depressive symptoms among healthcare workers. Huang et al. (41) also reported that supervisor support significantly moderated the link between work– family conflict and burnout among nurses in Taiwan, highlighting its cross-cultural relevance.

Taken together, these findings support the proposition that perceived supervisor support functions as a moderator that can attenuate the negative impact of work overload on patient safety behaviours. According to the JD–R model (35), it can serve as a vital job resource that reduces strain, fosters adaptive coping, and promotes optimal performance, particularly among vulnerable groups such as nursing interns. Thus, the following hypothesis is proposed:

*H2*: Perceived supervisor support moderates the relationship between work overload and patient safety behaviours, such that the positive association is weaker when supervisor support is high.

## Perceived coworker support as a buffer

Drawing on the JD-R model (19), this study also contends that perceived coworker support functions as a critical job resource that can mitigate the detrimental effects of work overload on patient safety behaviours. Coworker support refers to the instrumental assistance, advice, and emotional encouragement provided by colleagues within the workplace (42, 43). In high-pressure settings such as hospitals, support from peers plays a vital role in reducing occupational stress, enhancing job satisfaction, and promoting effective performance (44). For nursing interns, peer support may facilitate task sharing, foster knowledge exchange, and offer emotional reassurance, all of which are essential for managing excessive job demands (45).

Empirical evidence highlights the buffering role of coworker support in high-demand work environments. For instance, coworker support has been shown to reduce psychological strain and burnout associated with work overload (46). Qaiser et al. (47) found that coworker support moderated the relationships among work overload, negative affectivity, and work–family conflict in predicting emotional exhaustion among hospital staff. Similarly, Li et al. (48) reported that social support, including that from colleagues, attenuated the negative effects of job strain on depersonalization among operating room nurses. Chênevert et al. (49) demonstrated that colleague support moderated the impact of role stressors (e.g., role overload, conflict, and ambiguity) on change readiness in healthcare workers. Notably, Syed-Yahya et al. (18) observed that coworker support exerted a stronger mediating influence on safety performance than supervisory support, underscoring its significance in healthcare contexts.

Moreover, perceived social support from co-workers has been found to significantly enhance nurses' job performance, accounting for approximately 20% of the variance in reported performance levels (50). In collectivist cultures such as Nigeria (51), where interpersonal relationships and communal values are emphasized, coworker support may be particularly influential in helping nursing interns navigate occupational stressors. Given this cultural and empirical backdrop, it is anticipated that coworker support will buffer the relationship between work overload and patient safety behaviours. Therefore, the study proposes:

*H3*: Perceived coworker support moderates the relationship between work overload and patient safety behaviours, such that the positive association is weaker when coworker support is high.

## Materials and methods research design

This study employs a quantitative, cross-sectional survey research design to examine the relationships among the study variables. This design is appropriate because it allows the researcher to assess relationships among variables at a single point in time and to test moderation effects within organizational research.

## Participants and sampling

The target population for this study comprised nursing interns currently undergoing clinical placements in government teaching hospitals located in the Southwest region of Nigeria. A total of 250 nursing interns participated in the study. Of these, 65 (26.0%) were male and 185 (74.0%) were female. All participants satisfied the inclusion criteria, which required enrollment in a full-time nursing internship program and completion of at least three months of supervised clinical rotation under the guidance of registered nurses. Informed consent was obtained from all participants, and ethical approval was secured from the authorities of each participating hospital. Nursing interns were selected as the focus of this research due to their transitional status within the profession and the heightened risk of work overload they face in Nigeria's demanding clinical environments. A detailed summary of participants' socio-demographic characteristics is presented in Table 1.

**Table 1:** Descriptive statistics of socio-demographic variables (*N* = 250).

Socio-demographic variables	Frequency (n)	Percentage (%)
Gender		
Male	65	26.0
Female	185	74.0
Age group (years)		
20–24	98	39.2
25–29	132	52.8
30 and above	20	8.0
Duration of internship		
0–6 Months	118	47.2
7–12 Months	132	52.8
Current department		
Emergency	20	8.0
Intensive Care Unit (ICU)	30	12.0
Surgical	80	32.0
Pediatrics	38	15.2
Gynecological	46	18.4
Obstetrics	22	8.8
Orthopedics	6	2.4

A multi-stage sampling technique was employed to recruit participants. In the first stage, some public hospitals were purposively selected from government teaching hospitals located in the Southwest region of Nigeria to ensure representation across diverse healthcare settings. In the second stage, nursing interns within the selected hospitals were recruited using a convenience sampling method, based on their availability and willingness to participate at the time of data collection.

## Sample size

The sample size was determined using G*Power analysis for moderation analysis in regression, which suggests a minimum of 200–300 participants for adequate statistical power (0.80) at a medium effect size (*f*² = 0.15) and alpha level of 0.05. Thus, approximately 250–300 nursing interns were targeted.

## Measures

Data were collected through a structured self-report questionnaire, which comprised the following validated scales:

**Perceived Work overload:** was assessed using the 8-item scale developed by Cousins et al. (59). Participants responded to items on a 5-point Likert-type scale ranging from 1 (Never) to 5 (Always). A sample item is, ―I am pressured to work long hours. ‖ The original scale demonstrated good internal consistency (*α* = .83), and in the current study, the scale yielded a reliability coefficient of 0.87. Higher scores indicate greater perceived work overload, whereas lower scores reflect lower levels of work overload.
**Patient safety behaviours:** were assessed using an adapted version of the Medical Error Scale (MES) for nursing students, originally developed by Kahriman and Ozturk (56). The MES consists of 36 items across seven subscales: Falling, Blood and Blood Transfusion, Patient Transfer, Medication Administration, Communication, Infections, and Care Practices. In its original form, the MES used a 5-point Likert scale ranging from 1 (Never) to 5 (Always), with higher scores indicating fewer errors and greater adherence to patient safety protocols. The original scale demonstrated excellent reliability (*α* = .94). For the current study, the scale was modified to better suit the local context. The Likert scale was reversed (1 = Always to 5 = Never), so that higher scores reflected a higher frequency of patient safety-related errors or lower compliance with safety procedures. Face validation was conducted by a panel of Nigerian nurse educators and clinical experts, and minor linguistic adjustments were made to reflect locally used healthcare terminology. Construct validity was further supported by a pilot study involving nursing interns from three teaching hospitals. The adapted MES demonstrated strong internal consistency in this study (*α* = 0.81). Sample items include: ―I advise the patient's caregiver or relative (e.g., spouse, child, or sibling) to inform a nurse or ward attendant before leaving the patient unattended.‖ (Falling), ―I check the expiry date on the blood bag carefully before transfusion begins‖ (Blood and Blood Product Transfusion), and ―I confirm I understand the action, potential side effects, and interactions of any medication before administering it‖ (Medication administration).
**Perceived Supervisor Support:** was assessed using a 4-item scale from Rhoades et al. (60). Examples of items include ―My work supervisor cares about my well-being‖ and ―My supervisor is willing to help me when I need a special favour‖. Items are rated on a 5-point Likert format (1 = strongly disagree; 5 = strongly agree), with a Cronbach's alpha of 0.89 observed in the current study.
**Perceived Co-worker Support:** A 9-item scale from Ladd et al. (61) was used to assess the extent to which respondents believed their coworkers are supportive. Sample items were: ―My coworkers are willing to offer assistance or help me perform my job to the best of my ability‖ and ―My coworker really cares about my well-being.‖ In the current study, a Cronbach's alpha of 0.85 was obtained for the scale.

## Procedure

Participant recruitment was conducted in collaboration with hospital administrators and internship coordinators across selected hospitals. These key personnel assisted in the distribution of both online and paper-based questionnaires during scheduled training sessions for nursing interns. Prior to participation, all interns were provided with detailed information regarding the purpose of the study, and the voluntary nature of their involvement was clearly emphasized. Informed consent was obtained from each participant before the commencement of the survey. Anonymity and confidentiality were strictly maintained throughout the data collection process. Participants were assured that their responses would be used solely for research purposes and that there would be no personal or professional consequences associated with their participation. Data collection was carried out over a six-week period. During this time, completed questionnaires were retrieved either immediately in the case of paper-based surveys or via a secure online platform. No financial incentives or compensation were offered. Of the 300 nursing interns invited to participate, 250 provided usable responses, yielding a valid response rate of 83.3% after excluding incomplete submissions

## Data analysis

Data analysis was conducted using Statistical Package for Social Sciences (SPSS) version 27 and PROCESS Macro by Hayes for moderation analysis. Descriptive statistics was used to describe demographic characteristics and the distribution of study variables (means, standard deviations, frequencies). Cronbach's alpha was calculated to assess the internal consistency of the scales. A reliability coefficient of 0.70 and above was considered acceptable. Pearson's correlation coefficients were used to examine the relationships among work overload, patient safety behaviours, supervisor support, and coworker support. Moderation analysis was conducted using Hayes' PROCESS macro (Model 1) to test whether social support (perceived supervisor support and coworker support) moderated the relationship between work overload and patient safety behaviours.

## Ethical considerations

Ethical approval was obtained from the Ethics Committee of Department of Pure and Applied Psychology, Adekunle Ajasin University, Akungba-Akoko, Ondo State, Nigeria (Approval number: EA30/02/2025). All participants provided informed consent prior to their inclusion in the study. Participation in the study was completely voluntary, with participants fully informed about the study's purpose, procedures, and potential risks and benefits.

## Results

### Descriptive statistics of socio-demographic and work characteristics

A total of 250 nursing interns participated in the study. The socio-demographic and work characteristics of the participants are summarized in Table 1. As shown in Table 1, the majority of respondents were female (74%), reflecting the gendered nature of the nursing profession in Nigeria. Most participants were aged 25–29 years (52.8%), followed by those 20–24 years (39.2%). Only a small proportion was 30 years and above (8%). This aligns with the typical age range for nursing interns in Nigeria. Almost half of the participants had been on internship for 7– 12 months (52.8%), with the remainder having completed 0–6 months (47.2%) of their internship at the time of data collection. Of the 250 nursing interns, the largest proportion were placed in the surgical department (32.0%), followed by the gynecological (18.4%), pediatric (15.2%), and ICU (12.0%) departments. Smaller proportions were assigned to the emergency (8.0%), orthopedic (8.8%), obstetric (3.2%), and other (2.4%) departments.

### Common method variance test

To assess potential common method variance (CMV), Harman's single-factor test was performed using principal component analysis on all measurement items. The analysis produced five factors with eigenvalues greater than 1. The first factor accounted for 32.47% of the total variance, which is below the 50% threshold (52), suggesting that CMV was unlikely to bias the study results.

### Descriptive statistics and reliability coefficients of key variables

The preliminary analysis involved descriptive statistics and reliability tests among the study variables. These analyses were conducted to examine the distribution of the data and assess the internal consistency of the measurement scales. The results are presented in Table 2.

**Table 2:** Descriptive statistics and reliability coefficients.

Variables	Mean (M)	Standard deviation (SD)	Cronbach's alpha (*α*)
Work Overload	3.76	0.82	0.87
Patient Safety Behaviours	2.14	0.95	0.81
Perceived Supervisor Support	3.41	0.79	0.89
Coworker Support	3.58	0.84	0.85

As shown in Table 2, nursing interns reported a moderately high level of work overload (M = 3.76, SD = 0.82), indicating that excessive workload is a common experience. Patient safety-related errors were reported at a relatively moderate to high frequency (M = 2.14, SD = 0.95), but the presence of errors remains a concern. Both supervisor support (M = 3.41) and coworker support (M = 3.58) were rated moderately high, suggesting that nursing interns perceive some degree of social support in their work environment. The reliability (Cronbach's alpha) for all scales exceeded the acceptable threshold of 0.70, indicating good internal consistency.

### Correlation analysis

The relationship between the study variables was tested using Pearson correlation statistics. The results are presented in Table 3.

**Table 3:** Correlation matrix.

Variables	1	2	3	4
1. Work Overload	1			
2. Patient Safety Behaviours	.42**	1		
3. Perceived Supervisor Support	−.28*	−.30**	1	
4. Perceived Coworker Support	−.25*	−.27*	.54**	1

* *p* < .05.

** *p* < .01.

Results in Table 3 showed that there is a significant positive correlation (*r* = .42, *p* < .01) between work overload and patient safety behaviours, indicating that as nursing interns experience higher workloads, the likelihood of patient safety-related errors increases. Work overload is negatively correlated with both supervisor support (*r* = −.28, *p* < .05) and coworker support (*r* = −.25, *p* < .05). This suggests that as perceived support increases, the feeling of being overloaded tends to decrease. Both supervisor support (*r* = −.30, *p* < .01) and coworker support (*r* = −.27, *p* < .05) are significantly and negatively associated with patient safety behaviours, indicating that higher perceived support relates to fewer patient safety errors. There is a strong positive correlation (*r* = .54, *p* < .001) between supervisor support and coworker support, showing that interns who perceive strong supervisor support also perceive high coworker support.

### Hypotheses testing

Prior to conducting the moderation analysis, the assumptions of regression were assessed. Normality of the residuals was examined using skewness and kurtosis, which were within acceptable limits (±2) for all variables, indicating approximately normal distributions (53). Multicollinearity was assessed using variance inflation factors (VIF), and all predictors had VIF values below 3, indicating no significant multicollinearity (54). Moderation analysis was conducted using Hayes' PROCESS macro (Model 1) to test whether social support moderated the relationship between work overload and patient safety behaviours.

### Moderation by perceived supervisor support

Table 4 presents the Hayes PROCESS macro results of moderation analyses conducted using work overload as the predictor; perceived supervisor support as a potential moderator; and patient safety behaviours as outcome.

**Table 4:** Hayes PROCESS output on perceived supervisor support as a moderator in the relationship between work overload and patient safety behaviours.

Variables	*β*	SE	t	*p*	95% CI
Constant	1.85	0.32	5.78	.000	[1.22, 2.48]
Work Overload	0.46	0.09	5.11	.001	[0.28, 0.64]
Perceived Supervisor Support (PSS)	−0.29	0.11	−2.64	.009	[−0.51, −0.07]
Work Overload × PSS	−0.21	0.07	−3.00	.003	[−0.35, −0.07]
*R²*	0.31				
*F*	12.42, *p* < .001				

**Figure 1:**
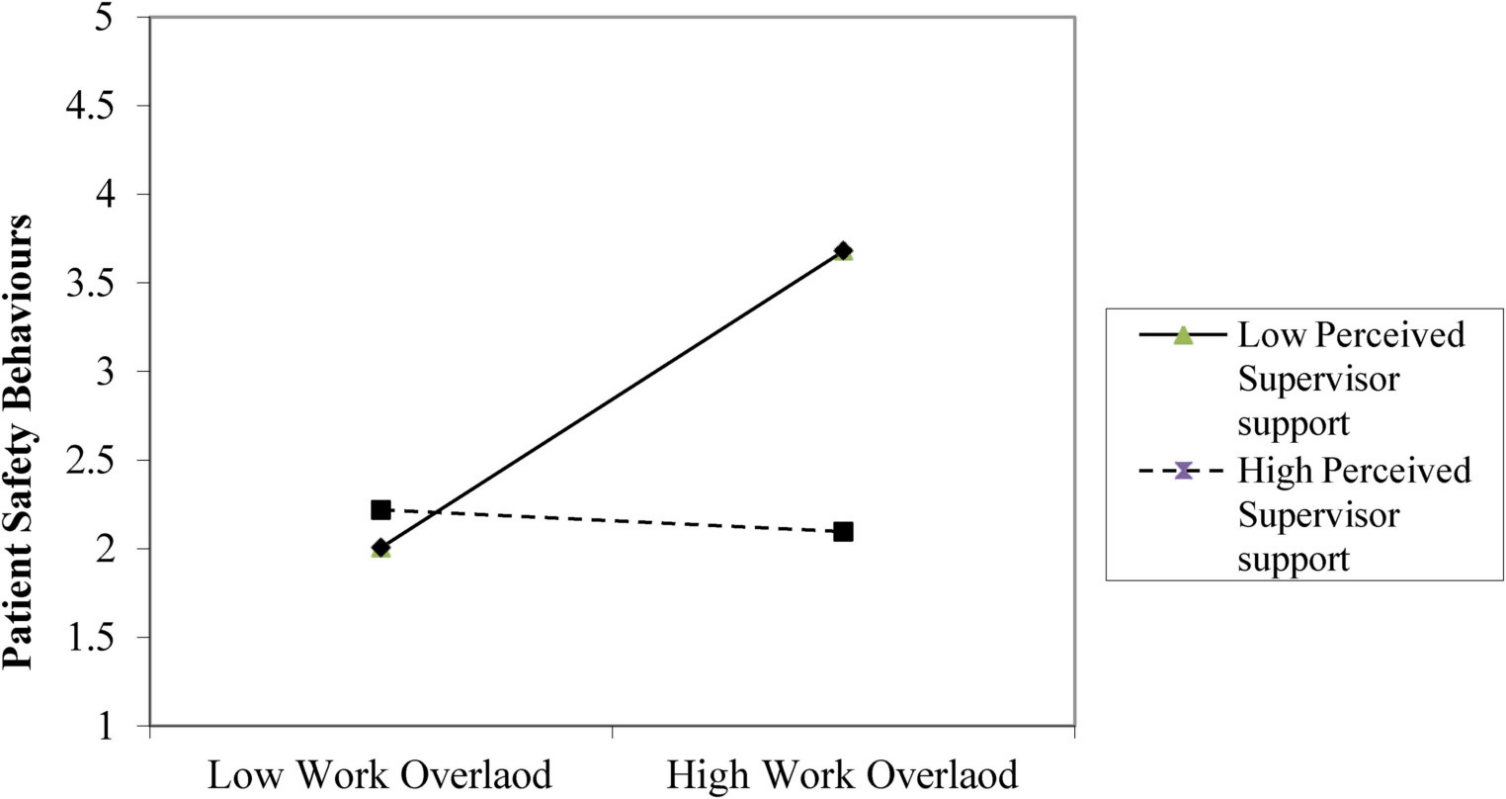
Interaction of work overload and perceived supervisor support on patient safety behaviours. This Figure showed that higher perceived coworker support attenuates the positive relationship between work overload and patient safety behaviours.

Results in Table 4 indicated that work overload significantly predicts patient safety behaviours (*β* = 0.46, *p* < .001, SE = 0.09, 95% CI [1.22, 2.48). Perceived supervisor support negatively predicts patient safety behaviours (*β* = −0.29, SE = 0.11, *p* < .01, 95% CI [−0.51, −0.07). The interaction between work overload and perceived supervisor support is significant (*β* = −0.21, SE = 0.07, *p* = .003, 95% CI [−0.35, −0.07), indicating that supervisor support weakens the positive effect of work overload on patient safety errors. The model explains 31% (*R*^2^ = 0.31, F = 12.42, *p* < .001) of the variance in patient safety behaviours. This result is illustrated in Figure 1. The addition of the interaction term accounted for a Δ*R*^2^ = 0.03, which is a small-to- medium effect size. Assumptions of normality, homoscedasticity, and multicollinearity (VIF < 2) were met.

### Moderation by perceived coworker support

Table 5 presents the Hayes PROCESS macro results of moderation analyses conducted using work overload as the predictor; perceived co-worker support as a potential moderator; and patient safety behaviours as outcome.

**Table 5:** Hayes PROCESS output on perceived coworker support as a moderator in the relationship between work overload and patient safety behaviours.

Variables	*β*	SE	t	*p*	95% CI
Constant	1.92	0.31	6.19	.001	[1.30, 2.54]
Work Overload	0.43	0.09	4.78	.001	[0.26, 0.60]
Perceived Coworker Support (PCS)	−0.27	0.10	−2.70	.008	[−0.47, −0.07]
Work Overload × PCS	−0.23	0.08	−2.88	.004	[−0.39, −0.07]
*R²*	0.29				
*F*	11.33, *p* < .001				

**Figure 2:**
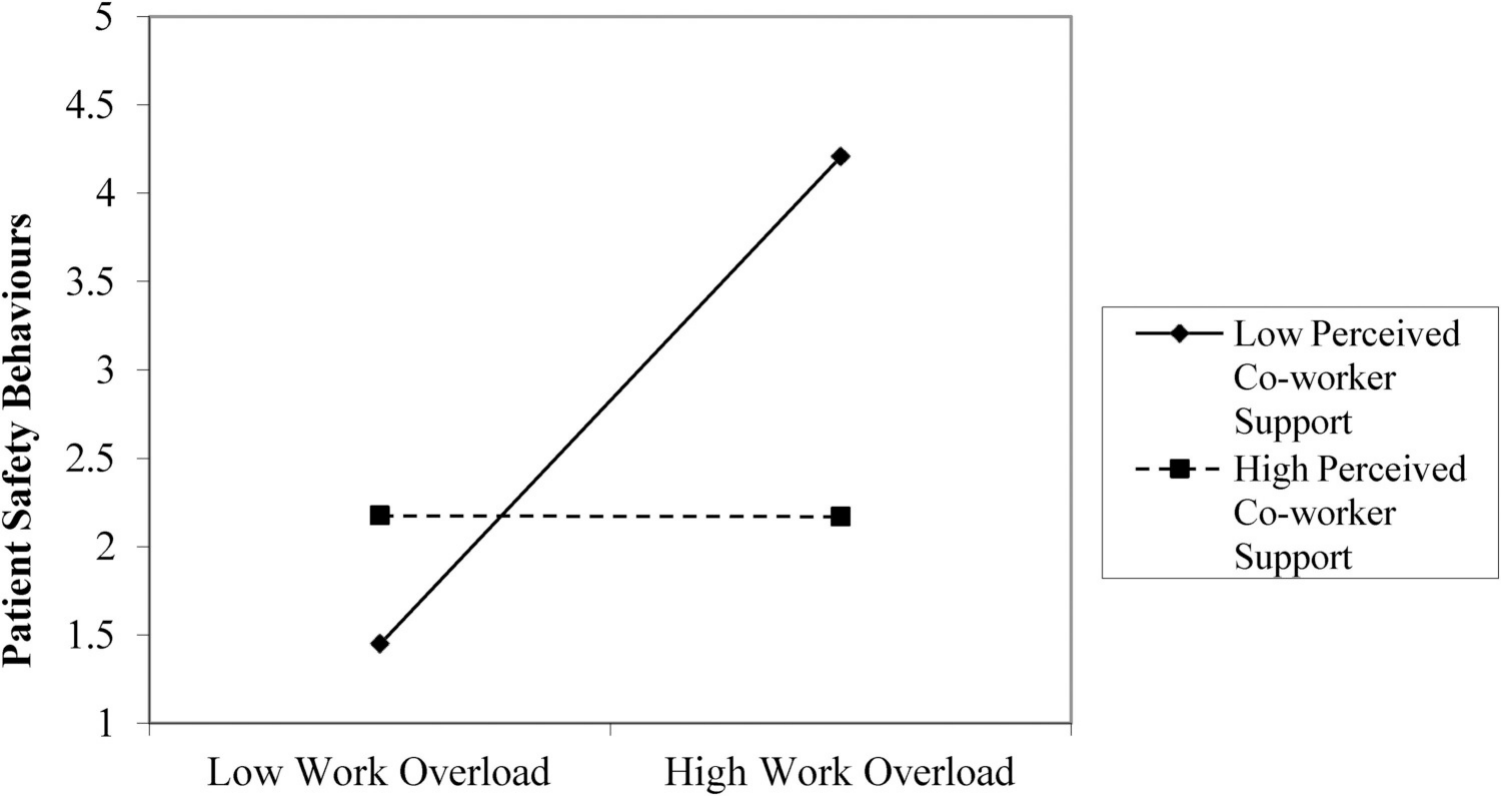
Interaction of work overload and perceived co-worker support on patient safety behaviours. This Figure showed that higher perceived supervisor support attenuates the positive relationship between work overload and patient safety behaviours.

Again, Table 5 revealed that work overload significantly predicts patient safety behaviours (*β* = 0.43, SE = 0.09, *p* < .001, 95% CI [0.26, 0.60). Perceived coworker support negatively predicts patient safety behaviours (*β* = −0.27, SE = 0.10, *p* < .001, 95% CI [−0.47, −0.07). The interaction between work overload and perceived coworker support is significant (*β* = −0.23, SE = 0.08, *p* = .004, 95% CI [−0.39, −0.07), suggesting that perceived coworker support reduces the impact of work overload on patient safety-related adverse events. The model explains 29% (*R*^2^ = 0.29, *F* = 11.33, *p* < .001) of the variance in patient safety behaviours. The result is illustrated in Figure 2. The addition of the interaction term accounted for a Δ*R*^2^ = 0.05, which represents a small-to-medium effect size. Assumptions of normality, homoscedasticity, and multicollinearity (VIF < 2) were met.

## Discussion

The present study investigated the relationship between work overload and patient safety behaviours among nursing interns, with a particular focus on the moderating roles of perceived supervisor and coworker support. The findings reveal several important implications for both theory and practice in healthcare settings.

Consistent with prior research [e.g., (11, 62)], the results demonstrate a significant positive correlation between work overload and patient safety-related errors. This result also aligns with previous studies indicating that excessive workload leads to cognitive fatigue, impaired decision-making, and diminished attention, all of which increase the likelihood of errors in healthcare settings (28, 63). This finding indicates that as nursing interns experience increased job demands, the likelihood of committing safety errors also rises. Given the high-stakes nature of clinical environments, excessive workload may impair interns’ ability to maintain attention, make sound decisions, and adhere to patient safety protocols, factors that collectively elevate the risk of adverse outcomes. In the Nigerian context, where understaffing and resource constraints are prevalent, the impact of workload on errors is particularly acute.

Moreover, perceived supervisor support and coworker support were both significantly and negatively associated with patient safety-related errors. These results underscore the buffering role of social support within the workplace, echoing findings from previous studies that emphasize the protective effect of supportive work environments on employee well-being and performance (44, 55). Interns who feel supported by their supervisors and colleagues may be better equipped to cope with occupational stressors and more confident in seeking help or clarification when needed, thereby reducing the occurrence of errors.

Notably, both interaction effects were statistically significant. The interaction between work overload and perceived supervisor support indicates that supervisor support moderates the relationship between work overload and patient safety-related errors. Specifically, high levels of supervisor support appear to weaken the adverse effect of work overload, suggesting that supportive supervision can serve as a critical resource in mitigating the negative consequences of demanding workloads. For nursing interns in Nigeria, supportive supervisors may help clarify expectations, provide guidance, and reduce the psychological burden of workload, thereby minimizing patient safety errors. This finding is consistent with the JD-R model which contends that supportive supervision reduces stress, enhances coping abilities, and protects employees from the negative effects of high demands (64).

Similarly, the interaction between work overload and perceived coworker support reveals that coworker support also attenuates the impact of work overload on patient safety errors. This aligns with literature emphasizing the protective role of peer support in reducing stress and enhancing performance in demanding work environments (42, 65). In collectivist cultures like Nigeria, where social bonds and communal relationships are emphasized, coworker support may provide not only practical help but also emotional encouragement, which can reduce the likelihood of mistakes. This finding aligns with the JD-R model (19), which posits that job resources such as social support can buffer the negative effects of job demands on employee outcomes.

Importantly, this study contributes uniquely to the literature by providing empirical evidence from the Nigerian context, which has been underrepresented in research on nursing interns and patient safety. Unlike most prior studies conducted in high-resource settings, our findings highlight the critical role of social support in mitigating work overload under conditions of staff shortages, resource limitations, and high patient-to-nurse ratios. This context-specific insight underscores the importance of culturally and structurally sensitive interventions to enhance supervisor and coworker support, which may have a pronounced impact on patient safety in African healthcare environments.

Taken together, these results highlight the importance of fostering a supportive work environment for nursing interns. In high-pressure healthcare settings, where the risk of error has serious implications, organizational interventions that enhance supervisor and peer support may serve as effective strategies to safeguard patient safety and enhance clinical performance.

## Theoretical implications

The findings of this study contribute meaningfully to the theoretical understanding of occupational stress and performance within healthcare settings, particularly through the lens of the JD-R model (19). The significant positive relationship between work overload and patient safety-related errors among nursing interns lends further empirical support to the JD-R framework by demonstrating how excessive job demands can lead to negative performance outcomes in high-stakes environments. This study reinforces the model's proposition that elevated demands, in the absence of adequate resources, deplete individual energy and cognitive resources, thereby increasing the likelihood of task-related failures.

More importantly, the moderating effects of perceived supervisor and coworker support offer robust theoretical insights into how job resources can buffer the adverse effects of high demands. These findings extend the JD-R model by illustrating that not all social support is equally protective; both hierarchical (supervisor) and lateral (coworker) support independently and significantly attenuated the relationship between work overload and patient safety-related errors. This highlights the multidimensional nature of job resources and suggests that different sources of support may play complementary roles in sustaining performance under pressure.

Additionally, the study contributes to the growing literature on error prevention in clinical education and early-career healthcare professionals. While previous research has predominantly focused on seasoned staff, these results underscore the importance of examining stress-performance dynamics among interns and trainees, who are particularly vulnerable to the pressures of work overload. The theoretical implication is that early interventions aimed at enhancing perceived support during the formative stages of professional development may have long-term benefits for both individual competency and organizational safety outcomes.

Overall, this study enhances existing theoretical models by empirically validating the buffering role of social support and advocating for a more nuanced understanding of how interpersonal dynamics shape the consequences of occupational stressors in healthcare settings.

## Practical implications

The findings of this study offer several important practical implications for healthcare institutions, nursing educators, and policymakers concerned with improving patient safety and promoting well-being among nursing interns. First, the significant positive association between work overload and patient safety behaviours highlights the urgent need to address excessive workload demands in clinical training environments. Hospital administrators and nurse managers should evaluate staffing levels, shift assignments, and task distribution to ensure that nursing interns are not overburdened beyond their capacity. Implementing workload monitoring systems and rotating high-demand tasks may help prevent cognitive fatigue and reduce the likelihood of errors.

Second, the protective roles of perceived supervisor and coworker support suggest that strengthening interpersonal support systems in healthcare settings can mitigate the negative effects of job stressors. Nursing supervisors should be trained not only in clinical oversight but also in providing emotional and professional support to interns. Regular check-ins, mentorship programs, and open-door communication policies may enhance interns' perceptions of supervisor support. Likewise, fostering a collaborative and team-oriented work culture can promote peer support, allowing interns to share concerns, seek assistance, and learn from more experienced colleagues without fear of judgment.

Third, these findings underscore the value of integrating support-building strategies into nursing education and orientation programs. Structured onboarding processes that pair interns with experienced preceptors, promote peer networking, and include stress management components can equip new professionals with the resources they need to navigate challenging work environments effectively.

Finally, from a broader organizational perspective, investing in supportive leadership practices and cultivating a psychologically safe work climate may not only improve intern performance but also lead to long-term benefits in staff retention, job satisfaction, and patient care quality. By recognizing and reinforcing the critical role of social support in clinical settings, healthcare institutions can create resilient systems that safeguard both their workforce and the populations they serve.

## Limitations and suggestions for future research

While the findings of this study provide valuable insights into the relationship between work overload, perceived support, and patient safety behaviours among nursing interns, several limitations should be acknowledged.

First, the cross-sectional design of the study limits the ability to draw causal inferences. Although significant associations were found, the directionality of the relationships cannot be conclusively established. Future research should employ longitudinal or experimental designs to better understand the temporal dynamics and causal mechanisms linking work overload, support systems, and clinical performance outcomes. Second, the study relied exclusively on self- reported measures, which may be subject to common method bias and social desirability effects. For instance, participants may have underreported the frequency of patient safety behaviours or overestimated perceived support. To enhance validity, future studies should incorporate objective indicators such as supervisor-rated performance, organizational error reports, or observational data alongside self-report instruments. Third, the sample consisted solely of nursing interns, which may limit the generalizability of the findings to other healthcare professionals or more experienced nurses. While interns represent a critical and vulnerable group in clinical settings, future research should examine whether similar patterns hold among different categories of healthcare workers across various levels of experience and specialties. Fourth, the study did not account for other potentially influential variables such as personality traits (e.g., resilience, conscientiousness), coping strategies, or organizational factors like staffing ratios and shift duration. Future research could adopt a more comprehensive model that incorporates individual and contextual moderators to provide a deeper understanding of the conditions under which work overload leads to patient safety behaviours.

Finally, although both supervisor and coworker support were found to buffer the impact of work overload, the study did not explore the mechanisms through which these forms of support exert their protective effects. Future investigations could use qualitative or mixed- method approaches to explore how support is perceived, accessed, and utilized in high-pressure healthcare environments. In sum, addressing these limitations in future research can help to refine theoretical models, inform targeted interventions, and strengthen the evidence base for promoting safer and more supportive clinical training environments.

## Conclusion

This study revealed that nursing interns who perceived work overload are more likely to commit patient safety-related errors, underscoring the detrimental impact of excessive job demands on clinical performance. Importantly, both supervisor and coworker support emerged as protective factors, significantly weakening the adverse effect of work overload on safety error occurrence. These results contribute to the growing body of literature grounded in the JD-R model, reinforcing the critical importance of social support in mitigating occupational stress and enhancing patient safety. For healthcare organizations and training institutions, the findings emphasize the need to implement supportive supervisory structures and promote team cohesion to buffer the effects of high workload, especially for early-career professionals. By addressing work-related stressors and fostering supportive clinical environments, healthcare systems can enhance the well-being and effectiveness of nursing interns, thereby promoting safer, more reliable care for patients. Future research that builds on these findings will be essential to developing more targeted interventions and strengthening the foundations of a resilient healthcare workforce.

## Data Availability

The raw data supporting the conclusions of this article will be made available by the authors, without undue reservation.
